# F-actin coordinates spindle morphology and function in *Drosophila* meiosis

**DOI:** 10.1371/journal.pgen.1011111

**Published:** 2024-01-11

**Authors:** Benjamin W. Wood, Xingzhu Shi, Timothy T. Weil

**Affiliations:** Department of Zoology, University of Cambridge, Cambridge, United Kingdom; College de France CNRS, FRANCE

## Abstract

Meiosis is a highly conserved feature of sexual reproduction that ensures germ cells have the correct number of chromosomes prior to fertilization. A subset of microtubules, known as the spindle, are essential for accurate chromosome segregation during meiosis. Building evidence in mammalian systems has recently highlighted the unexpected requirement of the actin cytoskeleton in chromosome segregation; a network of spindle actin filaments appear to regulate many aspects of this process. Here we show that *Drosophila* oocytes also have a spindle population of actin that appears to regulate the formation of the microtubule spindle and chromosomal movements throughout meiosis. We demonstrate that genetic and pharmacological disruption of the actin cytoskeleton has a significant impact on spindle morphology, dynamics, and chromosome alignment and segregation during maturation and the metaphase-anaphase transition. We further reveal a role for calcium in maintaining the microtubule spindle and spindle actin. Together, our data highlights potential conservation of morphology and mechanism of the spindle actin during meiosis.

## Author summary

The actin cytoskeleton is a fundamental component of eukaryotic cells. During meiosis, cytoplasmic actin has been shown to aid in spindle positioning in order to enable asymmetric division. One largely unexplored role of actin is its contribution during the meiotic segregation of chromosomes. Previously thought to be primarily regulated by the microtubule cytoskeleton, the discovery of a population of actin within the meiotic spindle of mouse and human oocytes has opened an entirely new avenue of investigation within the field of meiosis. Here, we reveal in *Drosophila melanogaster* oocytes a novel population of meiotic actin. Utilising a combination of genetic and pharmacological approaches, we demonstrate the importance of this spindle actin in the regulation of microtubule spindle morphology and chromosomal segregation during the first metaphase-anaphase transition following egg activation. We further explore the potential input from calcium signalling at the spindle. We thus demonstrate great similarity of spindle actin morphologies and mechanisms when compared with starfish, *Xenopus* and mammals. This suggests a level of conservation that reflects the fundamental nature of meiosis itself.

## Introduction

Meiosis is a well studied and documented process that is essential to produce haploid gametes during sexual reproduction. In mammals, fetal oogonia initiate meiosis synchronously, arresting at prophase I and pausing in this state until sexual maturity, whereupon an oocyte or small subsets of oocytes are released periodically, and meiosis is resumed [1]. Nuclear envelope breakdown initiates this resumption, resulting in the formation of a bipolar spindle network as microtubules polymerize, capture chromosomes and then align them on the metaphase plate [2]. The spindle interacts closely with the cytoplasmic actin, which aids in the asymmetric positioning of the spindle adjacent to the cortex, enabling asymmetric cell division to leave a single oocyte containing the necessary maternal components [2–7]. Oocytes are arrested in metaphase II until fertilization and subsequent egg activation. An intracellular rise in calcium then triggers the completion of meiosis, resulting in the formation of the maternal pronucleus which undergoes fusion with the paternal pro-nucleus to form a diploid zygote [8,9].

In *Drosophila melanogaster*, the process is similar but with a few differences. Oocytes are generated continuously when environmental conditions are favorable. Connected to supporting cells via cytoplasmic bridges and surrounded by a mono-layer of epithelial cells, the oocyte passes through 14 morphologically distinct stages during oogenesis. Meiosis is held in late prophase I for the majority of oogenesis, until the germinal vesicle breakdown (GVBD) (often used interchangeably with nuclear envelope breakdown) and the prophase to metaphase transition is completed [10–12]. Key features of this step can be observed within the *Drosophila* oocyte, such as GVBD and formation of a bipolar spindle as microtubule spindles capture the meiosis I chromosomes [13,14]. The spindle is a comparatively small structure in relation to the rest of the oocyte and lies parallel to the cortex at the dorsal-anterior tip of the oocyte, just below the dorsal appendages.

Unlike mammals and most vertebrates, the final meiotic arrest in *Drosophila* oocytes is at metaphase I, and the bipolar spindle structure that forms is more defined and has more focused spindle poles when compared to the “barrel” shaped spindles of mammals. Activation in *Drosophila* occurs prior to fertilization as the oocyte passes into the oviduct, which results in a calcium influx through transient receptor potential melastatin (TrpM) ion channels in the plasma membrane of the oocyte [15,16]. This calcium event enables the resumption of meiosis from its arrested state and can be observed using a variety of microtubule labelling tools [17].

Detailed cytological studies of *Drosophila* oocytes revealed that at the metaphase I arrest, the chromosomes exist as a central mass [18], including the non-exchange chromosomes, which can be visible as a separate entity during pro-metaphase or anaphase [17–19]. The metaphase to anaphase transition during egg activation is described as a stereotypical series of events that leaves spindle elongated and perpendicular to the cortex [20].

The mature *Drosophila* oocyte itself is approximately 500 μm in length, compared to the spindle which is approximately 10 μm (~50:1 ratio of oocyte to spindle). In contrast, the mouse oocyte has a ratio of approximately 5:1 oocyte to spindle length [21]. The *Drosophila* oocyte itself is vitellogenic and surrounded by protective outer casings, reflecting the need for the future embryo to be as robust as possible as they ultimately develop externally to the organism. These features can initially make distinguishing the components of the *Drosophila* spindle challenging. However, with a variety of genetic tools available, visualization and manipulation of spindle components can now be achieved, highlighting *Drosophila* as an important model system for understanding and future research of this field.

It has been observed that a population of actin exists within the mammalian oocyte that forms a spindle-like structure and has been shown to regulate chromosome alignment and segregation [21]. Treatment of these oocytes with cytochalasin D (cytoD) and knockdown of Formin-2, a key nucleator of spindle-like actin in mice, results in the misalignment of chromosomes during metaphase I and chromosome segregation errors during anaphase, often resulting in aneuploidy. Actin was shown to regulate chromosomal movements in part due to control of the kinetochore microtubules (K-fibres), indicating a likely role of actin in microtubule organization more generally [21]. Multi-color 3D-fluorescence microscopy revealed that human oocytes display a population of spindle actin similar to the population in mice, and additionally demonstrated that there is co-localization of γ-tubulin rich minus ends with filamentous actin clusters at the spindle poles [22]. Pharmacological manipulations revealed a co-operation of actin and microtubules at the meiotic spindle, as disruption of the microtubule spindle morphology is directly mirrored by changes to the spindle actin [22]. Taken together, this data suggests that the spatiotemporal organization of actin during oocyte maturation is similar to microtubule dynamics.

In this study, we utilize advanced imaging in conjunction with pharmacological and genetic manipulation to demonstrate that a population of spindle actin exists and appears to surround the microtubule spindle and chromosomes in the metaphase I arrested mature *Drosophila* oocyte. We show that the mammalian Formin-2 homologue, Cappuccino (Capu), is required for the formation of the spindle actin network, which undergoes strikingly similar morphological changes to the microtubule spindle during egg activation. Disruption of the actin cytoskeleton reveals a chromosomal phenotype that parallels that of a mammal: chromosome alignment and segregation appear disrupted. Whilst the actin cytoskeleton has been shown to be required for spindle positioning [23], such chromosomal segregation errors have not yet been explored in *Drosophila*, and these phenotypes may be attributed to the novel population of spindle actin demonstrated herein. Moreover, visualization and manipulation of calcium ions at this transition reveals the importance of calcium signaling for maintaining the morphology of the metaphase spindle and chromosome segregation. Taken together, our data suggests that actin is required upstream of the microtubules to regulate formation of the spindle.

## Results

### Actin is present at the spindle

**Fig 1:**
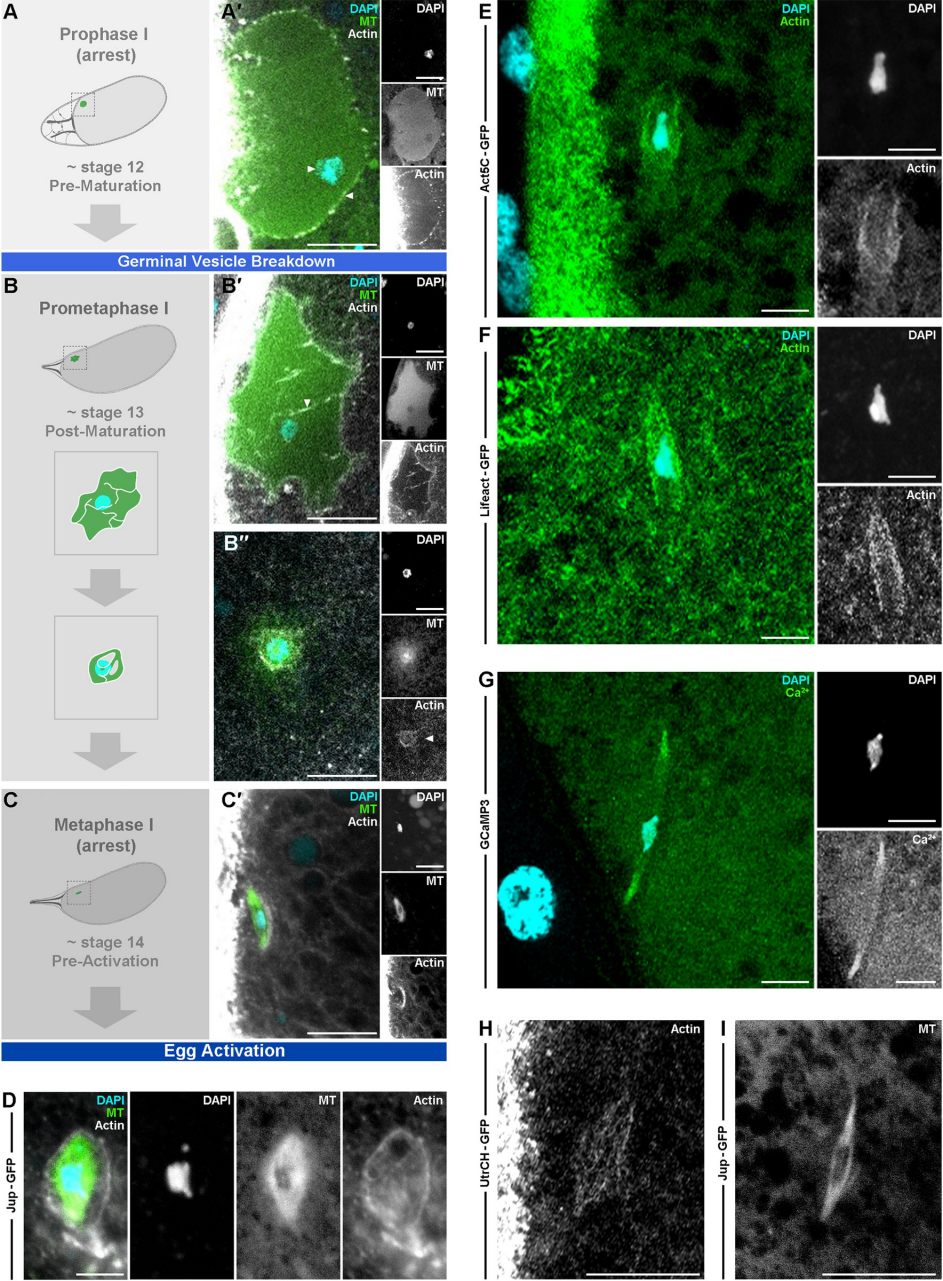
A spindle actin population is present in the metaphase-arrested *Drosophila* mature oocyte. **A-D.** Schematic and confocal Z-projection (10 μm) labeled for microtubules (Jup-GFP, green), actin (SiR-Actin, white), and DNA (DAPI, cyan). In stage 12 oocytes prior to egg maturation (**A,A′**) the DNA is condensed (white arrowhead, pointing right) and actin appears to enrich around the nucleus (white arrowhead, pointing left). (**A′**) n = 24 stage 12 oocytes and the depicted features were observed in 24 out of 24 samples (24/24). In stage 13 at maturation (**B-B′′**) actin spikes penetrate the nucleus (**B′**, white arrowhead) and then surround the DNA (**B′′**, white arrowhead actin inset) with the microtubule material appearing diffuse (**B′′**). (**B′)** n = 12/15 early stage 13 oocytes **(B′′**) n = 3/3 late stage 13 oocytes. In stage 14 metaphase I arrested oocytes (mature oocytes) (**C,C′**) the microtubule spindle forms and a population of actin, spindle actin, appears to associate with microtubules. Insets show induvial channels. (**C′)** n = 125/130 mature oocytes. Higher resolution image of early metaphase I arrest nucleus (**D**), actin appears to form a cage around the microtubules and DNA. In some cases, the 4th non-exchange chromosomes are visible as a smaller mass at each tip of the main body of chromosomes. **(D)** n = 56/60 mature oocytes. **E-F.** Confocal Z-projections (10 μm) labeled for actin (Act5C-GFP, green) (**E**), (Lifeact-GFP, green) (**F**), and DNA (DAPI, cyan) of a mature oocytes. The MI stage is marked by the spindle becoming parallel in orientation to the cortex. The cortical actin is visualized here as vertical green band of fluorescence between the oocyte nucleus and the follicle cell nuclei (far left) (**E**). The spindle actin is visible surrounding a central chromosomal mass using both markers (**E**, **F**). Insets show induvial channels, n > 15 mature oocytes per genetic actin marker. **G.** Confocal Z-projection (10 μm) labeled for Ca^2+^ (GCaMP3, green) and DNA (DAPI, cyan) of a MI oocyte. Higher calcium signal detected at the tip of the spindle pole, n = 15 mature oocytes. **H-I.** Confocal Z-projections (10 μm) from live time series labeled for actin (UtrCH-GFP) (**H**) or microtubules (Jup-GFP) (**I**) of MI oocytes. Similar structures and orientations are observed for actin and microtubule as compared to fixed samples. n > 37 mature oocytes per genetically encoded marker. Scale bar: 10 μm (**A-C**, **H-I**), 5 μm (**D-G**).

To test if the recently established novel population of actin within the spindle in mouse [21] and human [22] oocytes is conserved in *Drosophila*, we first sought to visualize actin and microtubules simultaneously in late-stage egg chambers. Using an endogenous F-actin stain (SiR-actin) and the microtubule binding protein Jupiter (Jup) fused to GFP (Jup-GFP), we show that prior to GVBD, the oocyte nucleus remains large and without clear microtubule filaments. An enrichment of actin is detected around the nuclear envelope and the DNA is condensed in a small area of the nucleoplasm (Fig 1A and 1A′). As the oocyte undergoes maturation and transitions from prophase I arrest to prometaphase I, we observe actin filaments forming around and throughout the nucleus as the nuclear membrane breaks down (Fig 1B and 1B′). As this stage progresses, we observe that a ring of actin forms around the DNA, as it continues to condense, while the microtubules appear to be mostly diffuse (Fig 1B′′). At the end of oogenesis, stage 14, when the mature oocyte is arrested in metaphase I, the microtubule spindles form an elliptical structure around the centrally lying chromosomes and what appears to be an outer ring of actin (Fig 1C and 1C′). Higher resolution imaging of the nucleus in a mature oocyte transitioning to the metaphase I shows a population of actin surrounding the DNA and microtubules as well as associating with the microtubule spindles (Fig 1D). These observations are reminiscent of an actin fishnet structure identified in starfish after GVBD [24,25].

To verify these findings, we used two well-established genetically encoded actin markers: Act5C-GFP [26], the actin 5C monomer conjugated to a GFP; and Lifeact-GFP [27], the actin binding protein Lifeact conjugated to a GFP. We again observe a distinct spindle actin population with the metaphase chromosomal mass located in the center (Fig 1E and 1F).

To further characterize this structure in the mature oocyte, we sought to observe calcium at this region. Previous work has shown a clear link between calcium and actin at *Drosophila* egg activation [28] and recent evidence in mature *Xenopus* oocytes highlights the presence of enriched calcium and necessity of localized calcium signaling at the spindle for regulation of microtubules [29]. Using GCaMP3 [30], a genetically encoded calcium sensor to visualize calcium *in vivo*, we observe an increased fluorescence intensity at the spindle indicative a calcium enrichment (Fig 1G).

Finally, we used UtrCH-GFP, the calponin-homology domain of Utrophin (Utr), required for the actin binding capacity of Utr, conjugated to GFP, to visualize actin in live samples. This tool has been used previously in other systems to successfully label the spindle actin for live analysis [21]. We once again show a highly distinct population of actin (Fig 1H) that resembles the microtubule spindle (Fig 1I), when visualized in live samples using Jup-GFP. The actin appears to mirror the microtubule elliptical shape with focused poles, a unique feature of the *Drosophila* meiotic spindle, which will henceforth be referred to as spindle actin.

### Spindle actin regulates metaphase microtubule spindle and chromosomes

**Fig 2:**
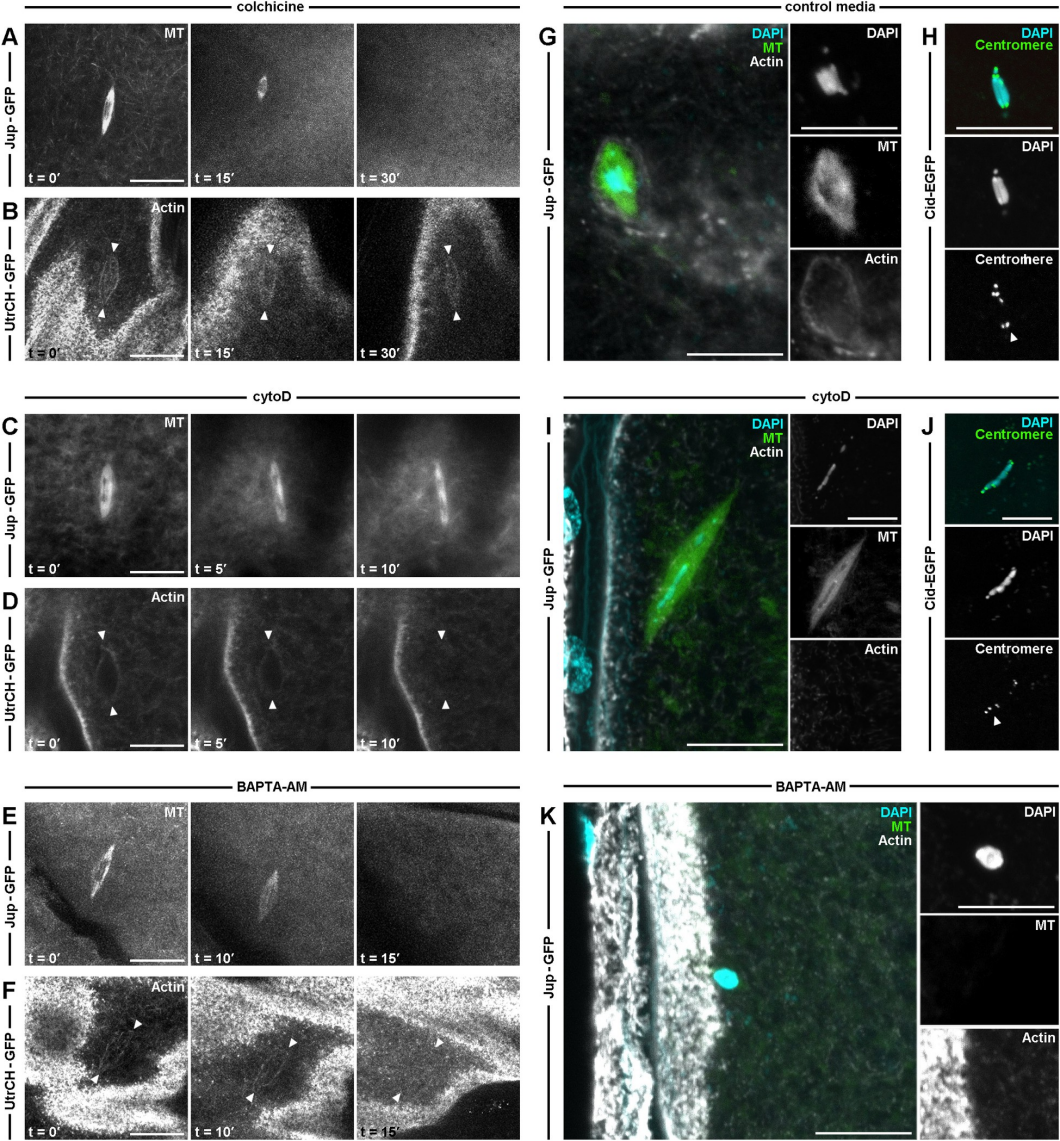
The actin cytoskeleton promotes maintenance of spindle microtubules and regulation of chromosomal alignment. **A-F.** Confocal Z-projections (10 μm) from live time series labeled for actin (UtrCH-GFP) or microtubules (Jup-GFP) of MI oocytes treated with colchicine (**A-B**), cytoD (**C-D**) and BAPTA-AM (**E-F**). Microtubules (**A**) first appear as a typical spindle structure (t = 0′) and depolymerize post colchicine treatment (t = 30′), whereas spindle actin (**B**, white arrowhead) is not affected. (**A-B**) n = 10 mature oocytes per genetically encoded marker treated. Before addition of cytoD (t = 0′), the microtubule spindle appears as a typical elliptical structure of approximately 10 μM (**C**). Post-cytoD treatment (t = 10′) the spindle has undergone a distinct morphological change as it elongates (**C**). Spindle actin (**D**, white arrowhead) appears parallel to the cortex before the addition of cytoD (t = 0′) and becomes depolymerized post treatment (t = 10′). (**C-D**) n = 9 mature oocytes per genetically encoded marker treated. Both microtubule spindle (**E**) and spindle actin (**F**, white arrowhead) depolymerize post BAPTA-AM treatment (t = 15′), suggesting calcium is required to maintain the metaphase spindles. (**E,F**) n = 8 mature oocytes per genetically encoded marker treated. **G-J.** Confocal Z-projections (10 μm) labeled for microtubules (Jup-GFP, green), actin (SiR-Actin, white), and DNA (DAPI, cyan) (**G,I**) or centromere (Cid-EGFP, green) and DNA (DAPI, cyan) (**H,J**) of MI oocytes incubated in control media (**G-H**) or cytoD (**I-J**). Spindle actin and the microtubule spindle appear to surround the chromosomes in MI oocytes incubated in control media (**G**). Centromeres are detected on each aligned chromosome (**H**, white arrowhead). (**G-H**) n = 40 mature oocytes per genetically encoded marker. Spindle actin depolymerizes post-cytoD treatment and the microtubule spindle is significantly elongated (**I**). Distance between centromeres also increases and the number of centromeres is unequally distributed between the two poles (**J**). (**I,J**) n = 50 mature oocytes per genetically encoded marker treated, (**I**) unpaired t-test, P value < 0.005. Insets show induvial channels (**G,H,K**). **K.** Confocal Z-projections (10 μm) labeled for microtubules (Jup-GFP, green), actin (SiR-Actin, white), and DNA (DAPI, cyan) of MI oocytes incubated in BAPTA-AM. A loss of the spindle and a round chromosomal mass in close proximity of the cortex are detected post-treatment. n = 10 mature oocytes. Insets show induvial channels. Scale bar: 10 μm (**A-K**).

To test the function of spindle actin, we first examined the relationship between the spindle actin and the microtubule spindle. Oocytes expressing markers for microtubules (Jup-GFP) or actin (UtrCH-GFP) were incubated in the microtubule depolymerizing agent colchicine (Fig 2A and 2B). As expected, we observe a complete loss of the microtubule spindle (Fig 2A), however, the spindle actin remained (Fig 2B). Next, depolymerization of the actin cytoskeleton using cytoD resulted in an elongated morphology of the microtubule spindle (Fig 2C), as well as the expected loss of spindle actin (Fig 2D). These data suggest that the spindle actin population is not dependent on the presence of the microtubule spindle filaments and that spindle actin could be important for organizing the microtubule spindle.

To test the role of calcium at the spindle, mature oocytes were incubated in the membrane permeable calcium chelating agent BAPTA-AM. The removal of calcium results in the loss of both the microtubule spindle (Fig 2E) and the spindle actin (Fig 2F), suggesting that calcium signalling is required to maintain the spindle apparatus but not showing that this is a direct effect. This is, however, similar to results in *Xenopus* in which BAPTA incubation caused microtubule depolymerization [29].

Considering the function of the microtubule spindle in chromosome segregation, we next asked if the spindle actin is involved in this process. First, we observed the difference in chromosome alignment between the metaphase-arrested spindle in control media (Fig 2G and 2H) compared to after disruption of actin with cytoD (Fig 2I and 2J). Separately using DAPI and a centromere marker, CENP-A homolog centromere identifier (CID) fused to an enhanced GFP (Cid-EGFP) [31], we show that there is alteration to the alignment as we detect multiple chromosomal masses spreading to either pole of the spindle axis (Fig 2G–2J). When analyzed, both DAPI and Cid show similar data for the spread of chromosomes and make us confident that DAPI is dependable to measure phenotypes. Moreover, BAPTA-AM incubations result in compaction of the chromosomes that closely associates with the oocyte cortex (Fig 2K). This phenotype is likely explained by the previous results in which BAPTA causes complete loss of the microtubules and actin within the spindle [29].

**Fig 3:**
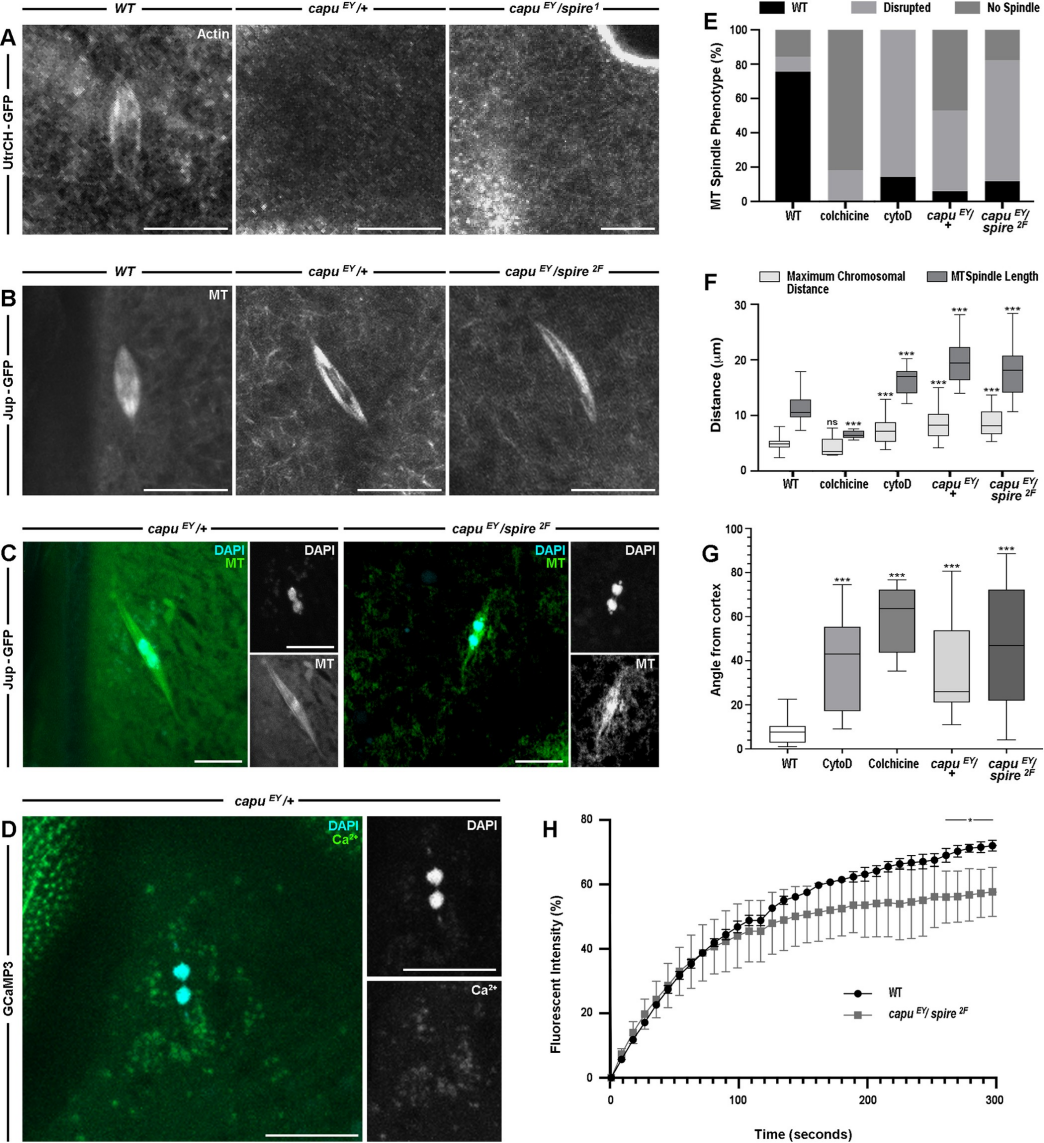
The spindle actin is required for recruitment and the position of the metaphase I spindle and to maintain chromosomal integrity. **A-B.** Confocal Z-projections (10 μm) from live time series of *WT*, *capu*^*EY12344*^*/*+, and *capu*^*EY12344*^*/spire*^*1*^ or *capu*^*EY12344*^*/spire*^*2F*^ in MI oocytes, labelled for actin (UtrCH-GFP) (**A**) or microtubules (Jup-GFP) (**B**). While the cortical actin (**A**, right panel, top right corner) could still be observed, spindle actin is not detected in the heterozygous or trans-heterozygous mutants. (**A**) n = 30 mature oocytes per genotype. Microtubules are elongated (**B**) and no longer parallel to the cortex in both the heterozygous and the trans-heterozygous mutants. (**B**) n = 30 mature oocytes per genotype. (Note: *capu*^*EY12344*^ referred to as *capu*^*EY*^ in panels in all figures). **C.** Confocal Z-projections (10 μm) labeled for microtubules (Jup-GFP, green) and DNA (DAPI, cyan) of *capu*^*EY12344*^*/*+ (left) and *capu*^*EY12344*^*/spire*^*2F*^ (right) in MI oocytes. DNA stains in both mutants reveal a separation of the chromosomal mass into two units that begin to migrate along the spindle axis. Insets show induvial channels. n = 20 mature oocytes per genotype. **D.** Confocal Z-projection (10 μm) labeled for calcium (GCaMP3, green) and DNA (DAPI, cyan) of a *capu*^*EY12344*^*/*+ MI oocyte. A loss of the calcium signal in a spindle shape and separation of the chromosomes into two separate masses are detected. Insets show induvial channels. n = 13 mature oocytes. **E-G.** Graphs summarising the proportion of microtubule spindle phenotype (**E**), maximum chromosomal distance and microtubule spindle length (**F**), and the angle of spindles from cortex (**G**), for *WT*, post-colchicine, post- cytoD, *capu*^*EY12344*^*/*+ and *capu*^*EY12344*^*/spire*^*2F*^ MI oocytes. The proportion of oocytes with a *WT* microtubule spindle phenotype are significantly decreased after drug treatment and in mutant backgrounds (**E**). Most microtubule spindles found in the experimental groups are either disrupted (elongated in cytoD and mutants or shortened in colchicine) or not detected. We do see a proportion of *WT* oocytes with no spindle detected, which is likely a technical issue due to the orientation of the oocyte or labelling efficiency. (**E**) n > 16 mature oocytes per treatment or genotype, Fishers Exact Test, P value <0.05. Comparison of the maximum chromosomal distance and the microtubule spindle length indicate a significant increase in cytoD treated and mutant backgrounds compared to *WT* (**F**). In the few mature oocytes where microtubule spindle where still detected 15 minutes after colchicine addition (n = 11 mature oocytes), the spindle length is reduced without any significant impact on the maximum chromosomal distance. (**F**) n >16 mature oocytes per treatment or genotype, student’s t-test, ***< 0.005. Comparison of the spindle-cortex angle (degrees) indicates a significant increase in cytoD, colchicine treated, and mutant background compared to *WT*. (**G**) n >16 mature oocytes per treatment or genotype, student’s t-test, ***< 0.005. **H.** Recovery of fluorescence intensity following photobleaching of microtubules in *WT* and *capu*^*EY12344*^*/spire*^*2F*^ mutant. *WT* and mutant oocytes initially show similar recovery dynamics, however, a significant difference between the mutant and *WT* is observed over time (after 270 s). n = 6 mature oocytes per genotype, student’s t-test, *< 0.05. Scale bar: 10 μm (**A-D**).

To further examine the function of spindle actin, we used genetics to disrupt the *Drosophila* homologue of mammalian Formin-2, Capu, which functions as part of the Capu-Spire actin nucleating complex (Fig 3A–3H) [32,33]. Homozygous *capu* mutants resulted in lethality, therefore we used several alleles to generate different heterozygous and trans-heterozygous backgrounds for analysis. Live visualization of actin showed that both a *capu* heterozygous and a *capu* / *spire* trans-heterozygous oocytes background was sufficient to disrupt the formation of this spindle actin (Fig 3A). We then observed microtubule spindle in various *capu* and *spire* mutant backgrounds and show that the microtubule spindle are significantly elongated or no longer form (Fig 3B, 3E and 3F).

Similarly, in c*apu* and s*pire* mutant combinations a clear disruption to the centrally congressed metaphase chromosomes can be observed (Fig 3C). Measuring this spread of the metaphase I chromosomes as the maximum chromosomal distance reveals a significant increase in those oocytes with a disrupted spindle actin (Fig 3C and 3F). In addition, analysis of the angle between the spindle and the nearest cortex reveals a significant change when actin is disrupted (Fig 3C and 3G). We also find that disruption of the spindle actin with a *capu* mutant results in a loss of enrichment of calcium at the spindle and multiple chromosomal masses (Fig 3D).

Finally, we used fluorescence recovery after photobleaching (FRAP) to test the dynamics of microtubule recruitment in *wild-type* and *capu* / *spire* trans-heterozygous oocytes (Fig 3H). When the spindle actin is disrupted, we observed a small but ultimately significant change in the recovery dynamics of the microtubule spindle. The failure of the spindle to recover fluorescence to a *wild-type* level, suggests the spindle actin population may play a role in the recruitment of microtubules to the spindle itself.

Taken together, our analyses reveal an important relationship between the spindle actin and microtubule spindle, as actin is required for accurate formation of the microtubule spindle and regulation of its morphology. This appears conserved with studies in mice and humans, in which the spindle actin has been shown to be required for formation of K-fibres and recovery of the spindle structure [21,22].

### Functional importance of the spindle actin during anaphase I

**Fig 4:**
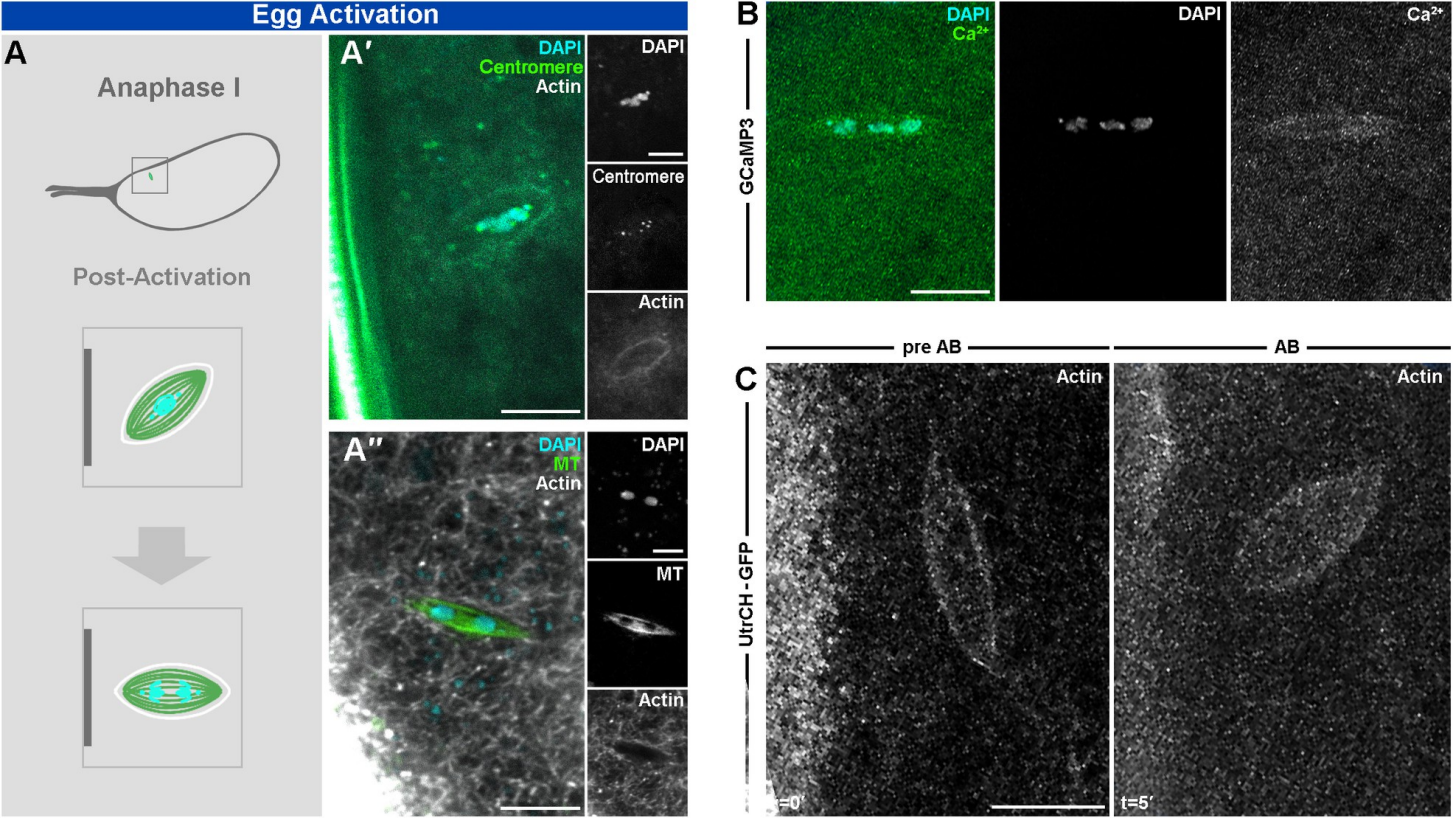
The spindle actin population is dynamic at egg activation. **A.** Schematic and confocal Z-projection (10 μm) labeled for centromere (Cid-EGFP, green) and DNA (DAPI, cyan) (**A′**) or microtubules (Jup-GFP, green), actin (SiR-Actin, white), and DNA (DAPI, cyan) (**A′′**) in early (**A′**) and later (**A′′**) anaphase I (AI) oocytes. Post egg activation, the chromosomes start to separate to opposite poles while the spindle first increases in width (**A′**) and then in length (**A′′**). The completion of AI is marked by the spindle becoming perpendicular in orientation to the cortex. Insets show induvial channels. (**A′**) n = 34/34 early activated mature oocytes. (**A′′**) n = 20/20 late activated mature oocytes. **B.** Confocal Z-projection (10 μm) labeled for calcium (GCaMP3, green) and DNA (DAPI, cyan) of an AI oocyte. Calcium signal spread evenly across the elongated spindle with separating chromosomal mass. n = 10 activated mature oocytes. **C.** Confocal Z-projections (10 μm) labeled for actin (UtrCH-GFP) from live time series of MI oocytes (left, t = 0′) incubated in activation buffer (AB) (right, t = 5′). Similar changes in shape and rotation observed as with fixed samples. n = 25 activated mature oocytes. Scale bar: 5 μm (**A-C**).

In order to test if the functional importance of this actin population extends throughout meiosis, we observed the spindle actin during egg activation. In *Drosophila*, egg activation occurs as the oocyte passes into the oviduct but can consistently be recapitulated *ex vivo* through incubation in a hypotonic buffer (activation buffer (AB)) [15,16,34]. This method was first established in the 1980s and has since been extensively utilized to understand the details of *Drosophila* egg activation. The first event of egg activation is swelling, which results in TrpM calcium ion channels opening and initiates a calcium transient to pass through the cell and trigger the metaphase-anaphase transition [15,16]. The Microtubule spindle undergoes a classical rearrangement at egg activation as the spindle initially elongates, then contracts and rotates in relation to the cortex, ultimately becoming perpendicular to the cortex [17]. Co-visualization with DAPI and centromeres, show the chromosomes associated in the center of the actin and expectedly perpendicular with the cortex (Fig 4A and 4A′). Our observation of microtubes and actin at egg activation reveals a similar morphological change for the spindle actin as it appears to contract and remain around the microtubule spindle (Fig 4A and 4A′). In addition, we observe a more even distribution of calcium at the spindle, no longer concentrated at the poles, after egg activation (Fig 4B). Importantly, we see a similar result with observing actin live during egg action (Fig 4C), thus reinforcing our conclusion that the interplay between spindle actin and the microtubule spindle continues in anaphase I.

**Fig 5:**
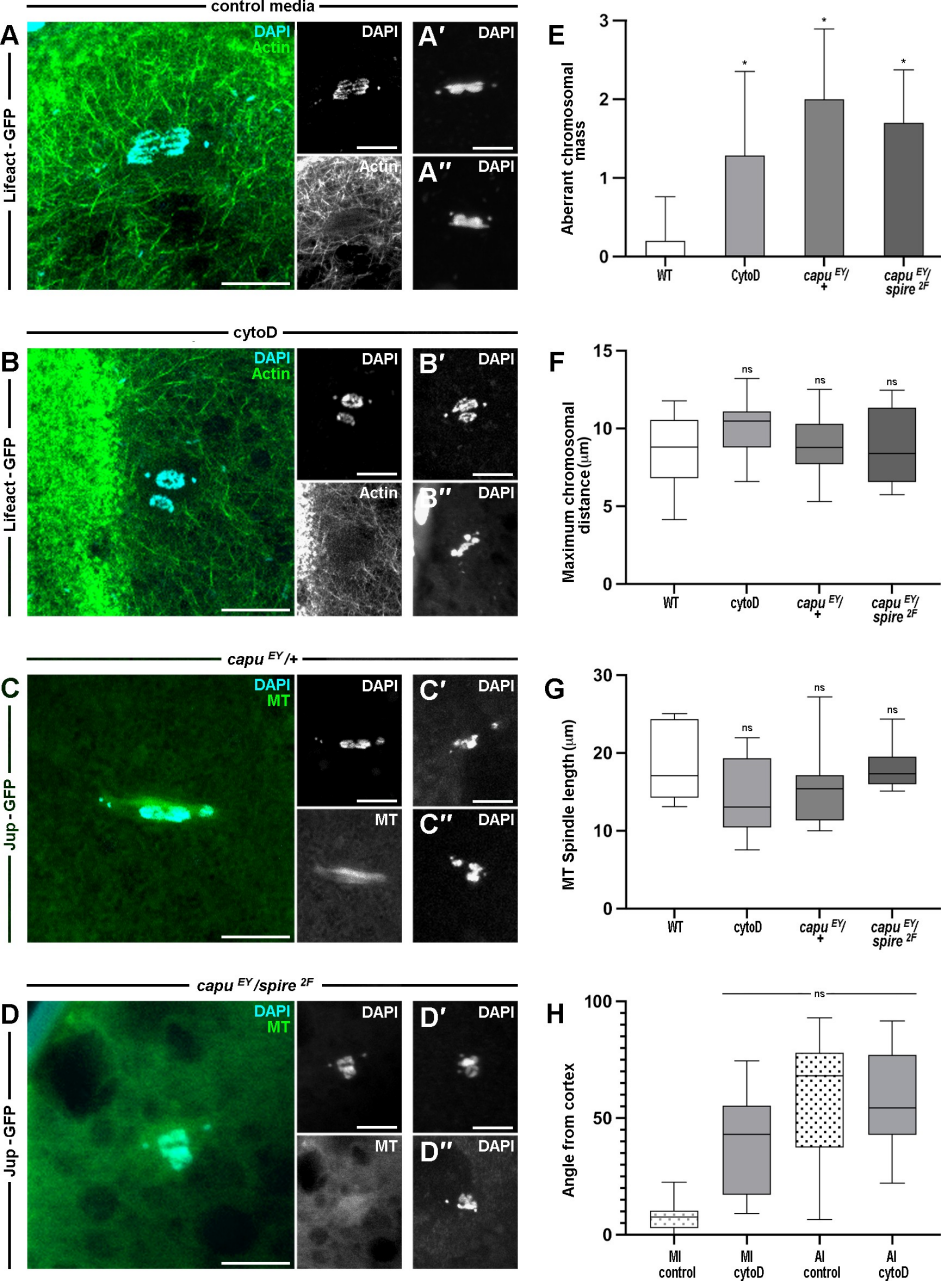
The spindle actin is required for accurate segregation of chromosomes during anaphase I. **A-B.** Confocal Z-projections (10 μm) labeled for actin (Lifeact-GFP, green) and DNA (DAPI, cyan) of AI oocytes incubated in control media (**A**) and cytoD (**B**). Spindle actin surrounds the segregating AI chromosomes in control media (**A**), but appears less defined than in MI oocytes, and is not detected after cytoD treatment (**B**). Chromosome segregation phenotype is disrupted in cytoD treated oocytes, many showing chromosomal units separated from the main mass (**B′-B′′**) compared to untreated controls (**A′-A′′**). Insets show induvial channels and additional chromosomal examples. n = 30 activated mature oocytes per treatment. **C-D.** Confocal Z-projections (10 μm) labeled for microtubules (Jup-GFP, green) and DNA (DAPI, cyan) of *capu*^*EY12344*^*/*+ (**C**) and *capu*^*EY12344*^*/spire*^*2F*^ (**D**) AI oocytes. Microtubule spindle are detected in both backgrounds, but chromosome segregation is misregulated. Examples of the variety of disrupted chromosome segregation phenotypes indicate chromosomal units separated from the main mass (**C′-C′′**, **D′-D′′**). Insets show induvial channels and additional chromosomal examples. n = 17 activated mature oocytes per genotype. **E-G.** Graphs summarising the number of aberrant chromosomal masses (**E**), maximum chromosomal distance (**F**), and microtubule spindle length (**G**) for *WT*, cytoD treated, *capu*^*EY12344*^*/*+, and *capu*^*EY12344*^*/spire*^*2F*^ AI oocytes. To quantify the aberrant chromosomal masses, we first defined the main chromosomal mass as the aligned materials in close association (< 1 μm), not including chromosome 4. Aberrant chromosomal masses were counted when material was disconnected from main mass by > 1 μm or when present > 1 μm outside of the spindle shape observed in wild type segregation. We also completed a 3D analysis to check the Z distance of > 1 μm from the main mass. Comparison of the number of aberrant chromosomal masses shows a significant increase in cytoD treated and mutant backgrounds compared to the *WT*. (**E**) n > 20 mature oocytes per treatment or genotype, student’s t-test, * < 0.05. Comparison of the maximum chromosomal distance and the microtubule spindle length shows no significant difference in cytoD treated and mutant backgrounds compared to the *WT*. (**F-G**) n > 30 mature oocytes per treatment or genotype, student’s t-test, not significant (ns). **H.** Graph summarising the angle of spindles from cortex before (left) and after (right) egg activation in *WT* and cytoD treated oocytes. Comparison of the angle between the spindle and cortex shows cytoD only has a significant effect on MI oocytes. There are no significant differences between the *WT* AI oocyte, and the cytoD treated MI and AI oocytes. n > 20 mature oocytes per treatment and stage, student’s t-test, not significant (ns). Scale bar: 5 μm (**A-D**).

We next observed actin at anaphase I and show that spindle actin remains around the separating chromosomes, perhaps less enriched than before egg activation (Fig 5A). Disruption of the anaphase spindle actin was achieved through addition of cytoD following AB treatment for 10 minutes to ensure oocytes had entered anaphase. This resulted in significant disruption to the segregation of the chromosomes, with frequent occurrence of aberrant chromosomal masses, which we define as chromosomes separate from the main axis of segregating chromosomes or a mass causing obvious non-uniformity (Fig 5B and 5E). In wild-type and non-disrupted anaphase oocytes the chromosomes separate as what appear visually as singular connected masses, except chromosome 4 which appear as small polar units. However, when the actin at the spindle is disrupted, such separating masses, which usually appear as a singular unit are disrupted to the extent that there appear to be several separating units, often not aligned with the rest of the separating chromosomes. This is a drastic phenotype, and one that can be visualised and quantified easily as such.

Similarly, visualization of the chromosomes in *capu* heterozygous and *capu* / *spire* trans-heterozygous backgrounds revealed microtubule abnormalities and aberrations in chromosome segregation (Fig 5C and 5D). Individual aberrant chromosomal masses were clearly identifiable and all oocytes tested demonstrated a significant increase in the number of these masses as compared to *wild-type* oocytes (Fig 5A). Furthermore, in the case of *capu* / *spire* trans-heterozygous oocytes, it was common to observe the 4th non-exchange chromosomes being closely located, suggesting a loss of spindle polarity (Fig 5D). This perhaps suggests a disruption to the bipolar spindle axis, as each of the non-exchange chromosomes would be expected to be at one pole of the spindle with the remaining chromosomes in-between.

Despite clear loss of accurate segregations, measurements of the maximum chromosomal distances and spindle length did not reveal any significant differences from control oocytes (Fig 5F and 5G). This may suggest that the chromosomes are still able to separate during anaphase, but with a loss of accuracy. In addition, we observed no significant difference in the angle of the spindle-cortex between cytoD treated oocytes in anaphase versus anaphase controls and cytoD treated metaphase oocytes (Fig 5H). We do, however, still observe a significant increase in the angle as compared to metaphase I controls. Together, this suggests that many anaphase events may still be capable of occurring when the spindle actin is disrupted. However, in the absence of spindle actin this appears to be brought about prematurely and results in a potential loss of organization, as we see dramatic aberrations in the quality of chromosome segregation.

## Discussion

This study establishes the existence of a population of spindle actin in close proximity with the microtubule spindle and chromosomes in *Drosophila* mature oocytes that appears to be required for the regulation of meiosis. Alteration of the actin through pharmacological or genetic disruption results in chromosome segregation errors, which may be attributable to the role of the spindle actin. We are aware that these approaches target all actin in the oocyte and are not specifically acting on the spindle population. For example, while we demonstrate the requirement of Capu in the formation of this spindle actin, one should consider that *capu and spire* have previously been shown to act together at stage 9 of oogenesis to organize the cytoskeleton which is required for axis formation [35,36].

At metaphase, the spindle lacks accurate chromosome alignment and congression, and at anaphase aberrant chromosomal masses are frequent. We also identify calcium as important in maintaining the spindle throughout metaphase. Together, we suggest that the spindle actin is required to mediate the accurate segregation of chromosomes through regulation of the microtubule spindle. Our data, together with recent work that reveals a population of spindle actin in mammals [21,22] and calcium enrichment in *Xenopus* [37], suggests there is a high level of evolutionary conservation at the spindle.

Prior to the formation of the spindle, we observed actin surrounding the nucleus reminiscent of the actin shell in mice oocytes [38], which aids the tearing and depolymerization of nuclear lamina during GVBD. In addition, our observation of the filamentous actin projecting through the nuclear envelope are similar to the spike-like structures in starfish oocytes [24]. To see whether this population of actin later forms transient fishnet structures that gather all chromosomes and coordinate their capture by microtubule spindles as in starfish oocytes [25], further exploration with higher resolution microscopy and better labelling tools are required.

The conservation, from mammals to *Drosophila*, we see in both functional and morphological similarities between spindle actin populations. Whilst variation is to be expected in comparison of meiotic mechanisms between species, such as final meiotic arrest occurring at metaphase I in *Drosophila*, in comparison to metaphase II in mammals, it appears that distinct populations of spindle actin may be another fundamental feature of meiosis. The potential conservation of this population of actin extends from its nucleation by the Formin-2 homolog Capu, to its functional role in the regulation of spindle microtubules and chromosomal movements.

However, there does appear to be some variation in the morphology and function of this spindle population. Much like the microtubule spindle, the spindle actin forms an elliptical shape with highly focused poles, unlike mammalian meiotic spindle structures which are more ‘barrel like’ in shape [2]. This population also seems to be resilient to disruption of the microtubule spindle, appearing to be important for recruitment of microtubules to the spindle and regulating overall spindle morphology, therefore suggesting there may be an upstream requirement of the actin cytoskeleton. When disrupted, either through disruption of *capu* or depolymerization by cytoD, significant defects in chromosome alignment can be observed. Observation of metaphase I oocytes indicated a spreading of the chromosomes along the metaphase spindle, with separation of chromosomes appearing reminiscent of pro-metaphase oocytes [19]. This could suggest a requirement of the spindle actin in the prophase-metaphase transition, a much earlier stage than has been observed in mammals to date. Perhaps this population of actin plays a more significant role in *Drosophila* meiosis I as mature oocytes arrest at metaphase I, which may indicate that the actin is important in meiotic arrest and release from this arrest during egg activation and onset of the metaphase-anaphase transition.

During egg activation, a global transient of calcium triggers a multitude of events, including global rearrangements of the actin cytoskeleton and resumption of meiosis [28]. There is building evidence that ties calcium and actin as two interlinked molecules in many signaling pathways; in *Drosophila* egg activation, dispersion of cortical actin enables entry of calcium in the form of a wave, which, downstream, effects a wave of reorganizing F-actin [28]. With many actin binding proteins (ABPs) being calcium sensitive, such as α-actinin and the villin family, and many calcium-sensitive proteins having downstream effects on the actin cytoskeleton, such as Calmodulin and calcineurin [39], it is possible that the calcium wave has a direct effect on the population of spindle actin, potentiating the release from meiotic arrest.

We have further observed what appears to be an enrichment of the calcium indicator GCaMP3 at the metaphase arrested spindle, suggesting an increased local concentration of calcium. Introduction of a calcium chelator results in depolymerisation of both the actin and microtubule spindle networks, indicating the likely requirement of calcium in maintenance of these populations, consistent with observations in *Xenopus* [29].

As demonstrated, disruption of Capu-Spire actin nucleating complex often results in the loss of formation of the microtubule spindle, indicating that the spindle actin may be required for recruitment of microtubules. This recruitment could be direct, however, there may be a contribution from the localized action of calcium. For example, microtubule associated proteins (MAPs), many of which are calcium sensitive and associate with both the actin and microtubule cytoskeleton [37], may mediate an actin-dependent recruitment of microtubules. Without the spindle actin, it is likely that the localization and coordination of these proteins are lost, resulting in a loss of calcium signaling at the spindle and concomitant loss of the microtubule spindle itself. Such mechanisms should be explored further in both *Drosophila* and mammals, as contributions from the spindle actin, microtubule spindle and calcium signaling are likely to demonstrate a degree of conservation.

## Materials and methods

### Fly maintenance

Fly stocks were raised on Iberian recipe fly food at 18°C, 21°C and 25°C. For dissection of mature oocytes, approximately 30 female flies with 5 male flies were transferred into a vial with Iberian recipe fly food and wet yeast for 48 hours at 25°C.

### Fly lines

*matα-GAL4*::*VP16*, *UASp-GCaMP3* [40]; *tub-GAL4VP16* (S. Roth); *UASp-Utrophin-CH-GFP/Tm3* [41]; *UASp-LifeactGFP/Tm3* (BL58717); *UASp-Act5CGFP/Tm3* (BL7309); *P{PTTGA}JupiterG00147* (BL6836); *P{PTT-un}CamP00695/CyO* (BL50843); *cid-EGFP*, *His2Av-mRFP* (BL91708); *capu*^*EY12344*^ [41]; *spire*^*2F*^*/CyO* (BL8723); *spire*^*1*^ (gift from I. Palacios).

### Preparing oocytes for live imaging

Ovaries from flies fattened for 48 hours on yeast were dissected onto a 22 by 40 mm cover slip, into series 95 halocarbon oil using forceps (11251–30 Dumont #5 forceps, Fine Science Tools) and a dissecting probe (0.25 mm straight 10140–01, Fine Science Tools) as described previously [42]. Mature oocytes were gently teased out of the ovaries and left for 10 minutes prior to imaging to allow them to settle onto the coverslip.

### Preparing *in vivo* activated eggs

Ovaries from flies fattened for 48 hours on yeast were carefully dissected onto a 22 by 40mm cover slip, such that the oviduct was still intact, into series 95 halocarbon oil. Oocytes were then gently teased out of the oviduct, carefully removing the surrounding oviduct tissues.

### Preparation of fixed samples

Ten to twenty ovaries were dissected from flies fattened for 48 hours on yeast into Schneider’s Insect Medium (Sch) (GibCo). Ovaries were splayed open, and oocytes gently teased out using fine forceps (11251–30 Dumont #5 forceps, Fine Science Tools) and a dissecting probe (0.25 mm straight 10140–01, Fine Science Tools). Mature oocytes were transferred into a 0.5mL eppendorf tube using a glass pipette. Sch was removed and 500 μL 4% paraformaldehyde (PFA) stabilized with phosphate buffer (Thermofisher Scientific) was added for 10–15 minutes on a rotary machine (PTR-35 360 vertical multi-function rotator, Thermofisher Scientific). Oocytes were then washed for 10 minutes, three times in 0.1% PBST (0.1% Triton X-100 (ThermoFisher Scientific) in PBS. Oocytes were then incubated for 2 hours in 1% PBST at room temperature with the following labelling probes: Alexa-Fluor Phalloidin 568, 1:500 (Molecular Probes); Alexa-Fluor Phalloidin 637, 1:500 (Molecular Probes); ChromoTek GFP-booster, 1:500 (Proteintech). When SiR-Actin is utilized, fixed oocytes were incubation for 40 minutes in 1% PBST with 1 μM SiR-Actin Probe 652 (Spirochrome) at 37°C. Oocytes were then washed, stained in glycerol and DAPI and mounted on a glass slide in Vectashield with DAPI (Vector Laboratories).

### *Ex vivo* egg activation

Mature oocytes were activated *ex vivo* through addition of the hypotonic, 260 mOsm, activation buffer (AB): 3.3 mM NaH_2_PO_4_, 16.6 mM KH_2_PO_4_, 10mM NaCl, 50 mM KCl, 5% polyethylene glycol (PEG) 8000, 2mM CaCl_2_, brought to pH 6.4 with a 1:5 ratio of NaOH:KOH [34]. Oocytes typically activate within two minutes of addition of AB. For fixation or drugs treatments, mature oocytes were activated with AB for 10 minutes before further processing.

### Pharmacological treatments

All pharmacological incubations were validated with either a Schneider’s Insect Medium (Sch) or PBS control and a DMSO control made to the appropriate dilution. Oocytes were incubated in Sch or PBS for 10 minutes whereupon samples were flooded with AB and visualized.

CytoD (Sigma Aldrich) was made to a final concentration of 2–20 μM in Sch or PBS. Oocytes were dissected into Sch or PBS a glass bottom dish. The Sch or PBS was carefully removed and replaced with the cytoD. Oocytes were incubated for 10 to 30 minutes in this solution prior to fixation or live imaging. Colchicine (Sigma Aldrich) was made to a final concentration of 50 μM in Sch or PBS. Oocytes were incubated for at least 30 minutes in this solution prior to fixation or live imaging, as above.

### Imaging with the Inverted Olympus FV3000 system

A 1.05 NA 30X silicone objective was used for whole oocyte imaging and a 1.35 NA 60X silicone objective for visualizing intracellular components. For high resolution imaging of the spindle, oocytes were oriented on the coverslip such that the dorsal appendages were in contact with the surface of the cover slip, therefore the dorsal side of the oocyte becomes the shallowest plane of visualization. Parameters for image collection were: 1.35 NA 60x silicon immersion objective, 10 μm Z-stack, 0.5 μm between each Z-slice, 1024×1024 pixels, approximately 15 seconds per stack.

### FRAP analysis

For FRAP of spindle components Jup-GFP was bleached for 10 seconds. Time lapse series of recovery was recorded every 5 seconds in single plane imaging of the cortex or every 30 seconds in Z-stack imaging of the spindle, both using the 488nm laser channel, 2 Airy unit pinhole, 1024x1024 pixels. For all FRAP series, background correction was performed by subtracting the fluorescence intensities of the unbleached cytoplasmic area from fluorescent intensities of bleached regions, with percentage fluorescence of the maximum plotted in graphs.
